# Transmit Power Allocation for Physical Layer Security in Cooperative Multi-Hop Full-Duplex Relay Networks

**DOI:** 10.3390/s16101726

**Published:** 2016-10-17

**Authors:** Jong-Ho Lee, Illsoo Sohn, Yong-Hwa Kim

**Affiliations:** 1 Department of Electronic Engineering, Gachon University, Seongnam 13120, Korea; jongho.lee@gachon.ac.kr (J.-H.L.); illsoo.sohn@gachon.ac.kr (I.S.); 2 Department of Electronic Engineering, Myongji University, Yongin 17058, Korea

**Keywords:** physical layer security, relay networks, full-duplex relay, power allocation, secrecy rate

## Abstract

In this paper, we consider a transmit power allocation problem for secure transmission in multi-hop decode-and-forward (DF) full-duplex relay (FDR) networks, where multiple FDRs are located at each hop and perform cooperative beamforming to null out the signal at multiple eavesdroppers. For a perfect self-interference cancellation (PSIC) case, where the self-interference signal at each FDR is completely canceled, we derive an optimal power allocation (OPA) strategy using the Karush-Kuhn-Tucker (KKT) conditions to maximize the achievable secrecy rate under an overall transmit power constraint. In the case where residual self-interferences exist owing to imperfect self-interference cancellation (ISIC), we also propose a transmit power allocation scheme using the geometric programming (GP) method. Numerical results are presented to verify the secrecy rate performance of the proposed power allocation schemes.

## 1. Introduction

To enable secure communication without being eavesdropped on by unintended receivers in wireless networks, physical layer security schemes exploit the physical characteristics of wireless channels with no need for upper-layer operations such as encryption techniques [1]. The rate at which a source can send information securely to an intended receiver is defined as the secrecy rate, and the maximum achievable secrecy rate is referred to as the secrecy capacity. It is known that we can achieve positive secrecy rates when the source-eavesdropper channel is a degraded version of the source-destination channel [2].

To obtain positive secrecy rates, even when the source-destination channel is worse than the source-eavesdropper channel, node cooperation has been extensively studied [3,4,5,6,7,8,9]. In node cooperation approaches, multiple relay nodes located between the source, destination, and eavesdroppers perform cooperative beamforming to enhance physical layer security. Three different operation modes have been suggested for cooperative relays, such as amplify-and-forward (AF), decode-and-forward (DF), and cooperative jamming (CJ) [3,6]. For the AF and DF modes, each relay receives the information signal from the source in the first time slot, whereas it cooperatively forwards the weighted version of its received signal for the AF mode and the weighted version of its re-encoded signal for the DF mode in the second time slot. For the CJ mode, the cooperative relays send weighted jamming signals to interfere with the eavesdropper. In [7], a two-way relay network formed by cooperative relays was considered, where some relays perform cooperative beamforming to receive and forward the signals from the sources, and other relays send jamming signals to confuse the eavesdropper. Furthermore, it was proven that secrecy rates can be further enhanced by using multiple DF relays that form a multi-hop relay network including more than two hops [10].

While the above-mentioned research considered conventional half-duplex relays (HDRs) where time partitioning was required for the transmission and reception of the relays, other research has utilized full-duplex operation that allows simultaneous transmission and reception on the same frequency [11,12,13]. In [11], the destination was designed to be a full-duplex receiver that receives the information signal from the source and transmits jamming signals to the eavesdropper simultaneously. In [12], a full-duplex relay (FDR) was considered in two-hop relay networks and two different full-duplex operation modes were suggested, such as full-duplex relaying and full-duplex jamming. Furthermore, in [13], relay nodes were designed to perform the full-duplex jamming mode in multi-hop relay networks that include more than two hops, where each FDR receives the information signal from the previous node and transmits jamming signals to the eavesdropper at the same time. As long as the self-interference signals induced by the full-duplex operation are properly suppressed by self-interference cancellation (SIC) schemes [14,15,16,17,18], the full-duplex approaches of [11,12,13] are shown to improve the physical layer security significantly.

It is noteworthy that the conventional studies for FDR networks in [12,13] assumed that a single FDR is located at each hop. In this study, we consider cooperative DF FDRs in multi-hop relay networks, where multiple FDRs equipped with a single antenna are located at each individual hop to perform cooperative beamforming to null out the signal at the eavesdroppers. Each FDR is assumed to operate in the full-duplex relaying mode to decode and forward the information signals at the same time instead of transmitting jamming signals. The transmit power allocation problem to maximize the achievable secrecy rate is formulated under an overall transmit power constraint to restrict the consumed power summed across the source and relays within a given limit. The power allocation problems for secure communication in relay networks are also found in [19,20,21]. While [19] takes into account a two-hop relay network with a single DF HDR, and a two-way relay network formed by a single AF HDR was considered in [20,21], our research considers a power allocation problem in a multi-hop relay network including more than two hops formed by multiple cooperative DF FDRs. We first considered a perfect self-interference cancellation (PSIC) case, where the self-interference at each FDR was perfectly canceled, and derived an optimal power allocation (OPA) strategy using the Karush-Kuhn-Tucker (KKT) conditions [22]. In the case where the residual self-interference signals remained due to the imperfect self-interference cancellation (ISIC), we also proposed a transmit power allocation scheme using the geometric programming (GP) method [22,23].

The remainder of this paper is organized as follows. Section 2 describes a signal model for multi-hop DF FDR networks considered in this research and describes the designs of a cooperative beamformer at each individual hop. In Section 3, we derive a transmit power allocation problem under the overall transmit power constraint and the DF relaying constraints. For the PSIC and ISIC cases, we solve the transmit power optimization problem using the KKT conditions and the GP method, respectively. Section 4 presents numerical results to verify the secrecy rate performance of the proposed power allocation schemes. Concluding remarks are provided in Section 5.

## 2. System Description

**Figure 1:**
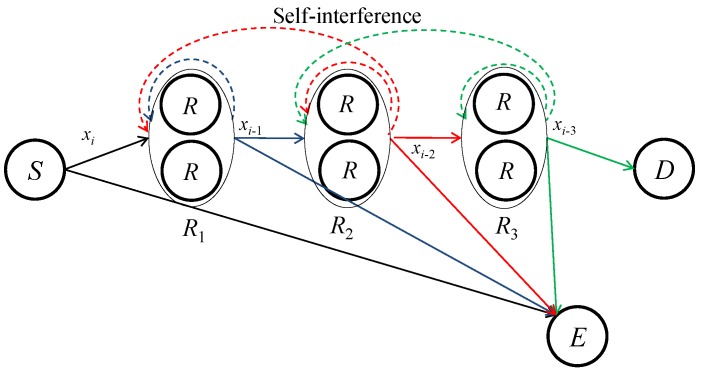
Illustration of a full-duplex multi-hop decode-and-forward (DF) relay network with N=3, $T_{1}=T_{2}=T_{3}=2$, and $T_{E}=1$.

As shown in Figure 1, we consider a wireless (N+1)-hop DF FDR network consisting of one source node *S*, trusted FDRs, one destination node *D*, and $T_{E}$ eavesdroppers. Let $R_{n}$ be a set of $T_{n}$ FDRs located at the *n*th relay position with n=1,2,⋯,N, which perform cooperative beamforming. Furthermore, we define *E* as a set of eavesdroppers. All nodes are assumed to be equipped with a single antenna. Each DF FDR decodes the signal from the previous adjacent nodes and forwards the weighted version of the re-encoded signal to the next adjacent nodes at the same time. The relays and *D* are assumed to receive the signal from the nodes located at their adjacent hops due to the propagation loss, whereas the eavesdroppers are assumed to overhear *S* as well as all the relays. For simplicity, we index *S* and $R_{n}$ by 0 and *n*, respectively. All channels are assumed to undergo flat fading. We let $h_{0,1}$ and $h_{N,D}$ denote $T_{1}\times 1$ and $1\times T_{N}$ complex channel vectors from *S* to $R_{1}$ and from $R_{N}$ to *D*, respectively. $H_{n,n+1}$ is defined as a $T_{n+1}\times T_{n}$ complex channel matrix from $R_{n}$ to $R_{n+1}$. Furthermore, $h_{0,E}$ is a $T_{E}\times 1$ complex channel vector from *S* to *E*, and $H_{n,E}$ is a $T_{E}\times T_{n}$ complex channel vector from $R_{n}$ to *E*. The noise at each node is assumed to be complex additive white Gaussian with zero-mean and variance $\sigma^{2}$.

### 2.1. Signal Model

In the *i*th time slot, *S* is assumed to send a data symbol $x_{i}$ to $R_{1}$. The FDRs in $R_{1}$ receive $x_{i}$ from *S* and send weighted versions of $x_{i-1}$ at the same time, which has been received and decoded in the (i−1)th time slot. In the same manner, the FDRs in $R_{n}$ send weighted versions of $x_{i-n}$ to $R_{n+1}$ and receive $x_{i-n+1}$ from $R_{n-1}$. Finally, the FDRs in $R_{N}$ send $x_{i-N}$ to *D* and receive $x_{i-N+1}$ from $R_{N-1}$. The received signals at the relays can be expressed as

(1) $$\begin{array}{ccc} y_{1} & = & \sqrt{P_{0}}h_{0,1}x_{i}+\underline{H_{1,1}w_{1}x_{i-1}}+\underline{H_{2,1}w_{2}x_{i-2}}+z_{1},\\ y_{n} & = & H_{n-1,n}w_{n-1}x_{i-n+1}+\underline{H_{n,n}w_{n}x_{i-n}}+\underline{H_{n+1,n}w_{n+1}x_{i-n-1}}+z_{n},\;n=2,3,\cdots ,N-1,\\ y_{N} & = & H_{N-1,N}w_{N-1}x_{i-N+1}+\underline{H_{N,N}w_{N}x_{i-N}}+z_{N}, \end{array}$$

where $x_{i}$ has unit power, $P_{0}$ is the transmit power of *S*, $w_{n}$ is a $T_{n}\times 1$ beamforming vector stacking the weights of the FDRs in $R_{n}$ with $w_{n}^{†}w_{n}=P_{n}$, ${\left(.\right)}^{†}$ denotes conjugated transpose, $P_{n}$ is the sum of the transmit powers of the FDRs in $R_{n}$, and $z_{n}$ is a $T_{n}\times 1$ additive white Gaussian noise (AWGN) vector at $R_{n}$. Note that the underlined terms in Equation (1) denote residual self-interferences after the SIC. The transmitted signal by the *t*th relay of $R_{n}$ induces its own self-interference and reaches the other FDRs in $R_{n}$ because they are located close to each other. Here, $H_{n,n}$ is a $T_{n}\times T_{n}$ complex channel matrix for the residual self-interference due to the transmission of $R_{n}$. In particular, the *t*th column of $H_{n,n}$ contains the complex channel gain for the residual self-interference induced by the transmission of the *t*th relay of $R_{n}$ for all the FDRs in $R_{n}$. Moreover, the transmission of $R_{n+1}$, originally destined for the next adjacent nodes, may reversely reach the FDRs in $R_{n}$ due to our assumption that the relays can hear nodes located at their adjacent hops. Keeping in mind that the FDRs in $R_{n}$ already know what the FDRs in $R_{n+1}$ have sent, we can also suppress these interferences using the conventional SIC schemes of [14,15,16,17,18]. $H_{n+1,n}$ in Equation (1) is the complex $T_{n}\times T_{n+1}$ channel matrix for the residual interferences at the FDRs in $R_{n}$ that are induced by the transmission of $R_{n+1}$. The received signals at *D* and *E* are given as

(2) $$\begin{array}{ccc} y_{D} & = & h_{N,D}w_{N}x_{i-N}+z_{D}, \end{array}$$

(3) $$\begin{array}{ccc} y_{E} & = & \sqrt{P_{0}}h_{0,E}x_{i}+\sum_{n=1}^{N}H_{n,E}w_{n}x_{i-n}+z_{E}, \end{array}$$

where $z_{D}$ and $z_{E}$ are the AWGN at *D* and *E*, respectively.

### 2.2. Cooperative Beamformer Design

Let us assume that global channel state information (CSI) is available because the eavesdropper is a legitimate user in the network and its transmission can be monitored [24]. In this scenario, the eavesdropper is assumed to be a low-level user able to access less information than the destination. In the case where only imperfect or partial CSI is available [25], we expect that our proposed scheme can be modified to cooperate with artificial noise-assisted techniques [26,27], where the spatial degrees of freedom provided by multiple cooperative relays at each hop are exploited to send artificially generated noise signals. In particular, for the residual self-interference channels, we assume that $H_{n,n}$ and $H_{n,n+1}$ are not available. If $H_{n,n}$ and $H_{n,n+1}$ are available, we can cancel even the residual self-interferences, which implies that the SIC is always perfect. Here, let us assume that only the residual self-interference powers are measurable.

Let us consider the design of $w_{n}$ to null out the signals at *E*. In order to determine $w_{n}$ with n=1,2,⋯,N−1, we incorporate the zero-forcing (ZF) approach in [3] with the max–min fair beamforming of [28] to null out the signals at *E* and maximize the minimum channel gain in $R_{n+1}$ shown as

(4) $${\bar{w}}_{n}=\underset{{\tilde{w}}_{n}}{\mathrm{argmax}}\min_{t=1,\cdots ,T_{n+1}}{\left|h_{n,n+1}^{\left(t\right)}\left(I_{T_{n}}-P_{n,E}\right){\tilde{w}}_{n}\right|}^{2},$$

where ${\bar{w}}_{n}^{†}{\bar{w}}_{n}=1$, $h_{n,n+1}^{\left(t\right)}$ is the *t*th row of $H_{n,n+1}$, $I_{T_{n}}$ is a $T_{n}\times T_{n}$ identity matrix, and $P_{n,E}$ is the orthogonal projection matrix onto the subspace spanned by $H_{n,E}$ given as [3]

(5) $$P_{n,E}=H_{n,E}^{†}{\left(H_{n,E}H_{n,E}^{†}\right)}^{-1}H_{n,E},$$

where ${\left(.\right)}^{†}$ denotes conjugated transposition. We can solve the optimization problem in Equation (4) by following the max-min fair beamforming approach of [28], which is highlighted in Appendix A. After obtaining ${\bar{w}}_{n}$, we compute $w_{n}$ as

(6) $$w_{n}=\sqrt{P_{n}}\frac{\left(I_{T_{n}}-P_{n,E}\right){\bar{w}}_{n}}{\left\|\left(I_{T_{n}}-P_{m,E}\right){\bar{w}}_{n}\right\|},$$

where n=1,2,⋯,N−1. Furthermore, we determine $w_{N}$ to null out the signals at *E* shown as [3]

(7) $$w_{N}=\sqrt{P_{N}}\frac{\left(I_{T_{N}}-P_{N,E}\right)h_{N,D}^{†}}{\left\|\left(I_{T_{N}}-P_{N,E}\right)h_{N,D}^{†}\right\|}.$$

Since all of the above beamformers null out the signals at *E*, each eavesdropper can receive the signal only from *S* and Equation (3) can be simply given as

(8) $$y_{E}=\sqrt{P_{0}}h_{0,E}x_{i}+z_{E}.$$

## 3. Transmit Power Allocation

In Equations (2) and (8), the rates at *D* and *E* are given as

(9) $$\begin{array}{ccc} R_{d} & = & \log_{2}\left(1+\alpha_{N,D}P_{N}\right), \end{array}$$

(10) $$\begin{array}{ccc} R_{e} & = & \log_{2}\left(1+\alpha_{0,E}P_{0}\right), \end{array}$$

where

(11) $$\alpha_{N,D}=\frac{{\left|h_{N,D}w_{N}\right|}^{2}}{\sigma^{2}},\;\;\alpha_{0,E}=\max_{t=1,\cdots ,T_{E}}\frac{{\left|h_{0,E}^{\left(t\right)}\right|}^{2}}{\sigma^{2}},$$

and $h_{0,E}^{\left(t\right)}$ is the *t*th entry of $h_{0,E}$. Using Equations (9) and (10), we compute the achievable secrecy rate as $R_{s}=\max\left\{R_{d}-R_{e},0\right\}$. In Equation (1), we compute the rate at the *t*th relay of $R_{n}$ given as

(12) $$\begin{array}{c} R_{n}^{\left(t\right)}=\log_{2}\left(1+\frac{\alpha_{n-1,n}^{\left(t\right)}P_{n-1}}{1+\beta_{n,n}^{\left(t\right)}P_{n}+\beta_{n+1,n}^{\left(t\right)}P_{n+1}}\right),\\ R_{N}^{\left(t\right)}=\log_{2}\left(1+\frac{\alpha_{N-1,N}^{\left(t\right)}P_{N-1}}{1+\beta_{N,N}^{\left(t\right)}P_{N}}\right), \end{array}$$

where n=1,2,⋯,N−1 and

(13) $$\alpha_{n-1,n}^{\left(t\right)}=\frac{{\left|h_{n-1,n}^{\left(t\right)}w_{n-1}\right|}^{2}}{\sigma^{2}},$$

(14) $$\beta_{n,n}^{\left(t\right)}=\frac{{\left|h_{n,n}^{\left(t\right)}w_{n}\right|}^{2}}{\sigma^{2}},\;\;\beta_{n+1,n}^{\left(t\right)}=\frac{{\left|h_{n+1,n}^{\left(t\right)}w_{n+1}\right|}^{2}}{\sigma^{2}}.$$

Here, $h_{n,n}^{\left(t\right)}$ and $h_{n+1,n}^{\left(t\right)}$ denote the *t*th row of $H_{n,n}$ and $H_{n+1,n}$, respectively. It is also meaningful to exploit a multi-antenna relay equipped with $T_{n}$ antennas at the *n*th relay position instead of $T_{n}$ single-antenna relays. In this case, Equation (12) should be modified for the rate at the multi-antenna relay, assuming that it performs maximal ratio combining [29].

In order to guarantee that each DF relay correctly decodes the information from the previous nodes and forwards it to the next nodes, we must consider the DF relaying constraints, where the rates at the relays are greater than or equal to $R_{d}$, (i.e., $R_{n}^{\left(t\right)}\geq R_{d}$). Under an overall power constraint *P*, we derive the optimization problem for transmit power allocation to maximize the achievable secrecy rate given as

(15) $$\begin{array}{c} \max_{P_{0},P_{1},\cdots P_{N}}\;R_{d}-R_{e}\;,\\ \;s.t.\;R_{n}^{\left(t\right)}\geq R_{d},\;\;t=1,2,\cdots ,T_{n},\;n=1,2,\cdots ,N,\;\\ \;\sum_{n=0}^{N}P_{n}\leq P,\;\\ \;0\leq P_{n}\leq P,\;n=0,1,\cdots ,N. \end{array}$$

We first consider the PSIC case where the self-interference is completely removed and derive the OPA using the KKT conditions. Then, the GP-based power allocation (GPPA) is also proposed for the ISIC case.

### 3.1. Optimal Power Allocation for PSIC

For the PSIC case, we have $\beta_{n,n}^{\left(t\right)}=\beta_{n+1,n}^{\left(t\right)}=0$ for all *t* and *n*. Substituting Equations (9), (10) and (12) into Equation (15), we rewrite the optimization problem in Equation (15) as

(16) $$\begin{array}{c} \min_{P_{0},P_{1},\cdots P_{N}}\;\log_{2}\left(1+\alpha_{0,E}P_{0}\right)-\log_{2}\left(1+\alpha_{N,D}P_{N}\right)\;,\\ \;s.t.\;\alpha_{N,D}P_{N}-\alpha_{n,n+1}P_{n}\leq 0,\;n=0,\cdots ,N-1,\;\\ \;\sum_{n=0}^{N}P_{n}\leq P,\;\\ \;0\leq P_{n}\leq P,\;n=0,1,\cdots ,N, \end{array}$$

where $\alpha_{n,n+1}=\min_{t=1,\cdots ,T_{n+1}}\alpha_{n,n+1}^{\left(t\right)}$. In Appendix B, we have proven the following:

- We can achieve positive secrecy rates only when $\alpha_{0,1}>\alpha_{0,E}$, and the OPA is given by
  (17) $$P_{n}=\frac{\gamma }{\alpha_{n,n+1}}P,\;\;P_{N}=\frac{\gamma }{\alpha_{N,D}}P,$$
  where n=0,1,⋯,N−1 and
  (18) $$\gamma =\frac{1}{\sum_{n=0}^{N-1}\frac{1}{\alpha_{n,n+1}}+\frac{1}{\alpha_{N,D}}}.$$
- We have no choice but to obtain zero secrecy rates when $\alpha_{0,1}\leq \alpha_{0,E}$.

Note that the OPA for the PSIC case is given in a simple closed form. As discussed in Appendix B, the OPA in Equation (17) satisfies the overall power constraint and the DF relaying constraints with equality, which depend only on the channel conditions between *S*, $R_{n}$, and *D* (i.e., $\alpha_{n,n+1}$ with n=0,1,⋯,N−1 and $\alpha_{N,D}$). It is noteworthy that the channel conditions for *E* (i.e., $\alpha_{0,E}$) only influence whether positive secrecy rates can be achieved or not.

### 3.2. GP-Based Power Allocation for ISIC

Now, let us consider that the residual self-interference exists due to the ISIC, which implies that $\beta_{n,n}^{\left(t\right)}$ and $\beta_{n+1,n}^{\left(t\right)}$ are non-zero. Substituting Equations (9), (10) and (12) into Equation (15) with $\beta_{n,n}^{\left(t\right)}$ and $\beta_{n+1,n}^{\left(t\right)}$, we obtain

(19) $$\begin{array}{ccc} \max_{P_{0},P_{1},\cdots P_{N}}\; & & \frac{1+P_{N}\alpha_{N,D}}{1+P_{0}\alpha_{0,E}},\\ s.t.\; & & \frac{P_{n-1}\alpha_{n-1,n}^{\left(t\right)}}{1+P_{n}\beta_{n,n}^{\left(t\right)}+P_{n+1}\beta_{n+1,n}^{\left(t\right)}}\geq P_{N}\alpha_{N,D},\;t=1,2,\cdots ,T_{n},\;n=1,\cdots ,N-1,\\ & & \frac{P_{N-1}\alpha_{N-1,N}^{\left(t\right)}}{1+P_{N}\beta_{N,N}^{\left(t\right)}}\geq P_{N}\alpha_{N,D},\;\;t=1,2,\cdots ,T_{N},\\ & & \sum_{n=0}^{N}P_{n}\leq P,\\ & & 0\leq P_{n}\leq P,\;n=0,1,\cdots ,N. \end{array}$$

Since it is difficult to obtain the optimal solution of Equation (19), we propose a suboptimal approach to maximize the lower bound of the objective function, $\frac{P_{N}\alpha_{N,D}}{1+P_{0}\alpha_{0,E}}$ [23], which is equivalent to minimizing $\frac{1+P_{0}\alpha_{0,E}}{P_{N}\alpha_{N,D}}$. Then, we obtain

(20) $$\begin{array}{ccc} \min_{P_{0},P_{1},\cdots P_{N}}\; & & \frac{1}{\alpha_{N,D}}P_{N}^{-1}+\frac{\alpha_{0,E}}{\alpha_{N,D}}P_{0}P_{N}^{-1},\\ s.t.\; & & \frac{\alpha_{N,D}}{\alpha_{n-1,n}^{\left(t\right)}}P_{n-1}^{-1}P_{N}\left(1+\beta_{n,n}^{\left(t\right)}P_{n}+\beta_{n+1,n}^{\left(t\right)}P_{n+1}\right)\leq 1,\;t=1,2,\cdots ,T_{n},\;n=1,\cdots ,N-1,\\ & & \frac{\alpha_{N,D}}{\alpha_{N-1,N}^{\left(t\right)}}P_{N-1}^{-1}P_{N}\left(1+\beta_{N,N}^{\left(t\right)}P_{N}\right)\leq 1,\;t=1,2,\cdots ,T_{N},\\ & & \frac{1}{P}\sum_{n=0}^{N}P_{n}\leq 1,\\ & & 0\leq P_{n}\leq P,\;n=0,1,\cdots ,N. \end{array}$$

Note that Equation (20) is a GP problem and the solution can be obtained by the GP solver [30]. Since we have N+1 optimizing variables and $N+2+\sum_{n=1}^{N}T_{n}$ constraints, the complexity of solving Equation (20) is $O({\left(N+1\right)}^{3}\left(N+2+\sum_{n=1}^{N}T_{n}\right))$ [31]. The detailed complexity analysis for solving a GP problem can be found in [32,33]. We refer to the above power allocation strategy as the GPPA scheme.

## 4. Numerical Results

In this section, numerical results are presented to verify the secrecy rate performance of the proposed power allocation schemes. We assumed that *S*, $R_{n}$, and *D* are located in a line as in [3] and [6], whereas the eavesdroppers are located vertically away from the line. The path losses from $R_{n}$ to their adjacent nodes were assumed to be almost the same, considering that the distances between the FDRs in $R_{n}$ themselves are much smaller than the distances between $R_{n}$ and their adjacent nodes. Similarly, it was also assumed that the eavesdroppers were closely located and the path losses from the other node to *E* are almost the same. The *S*-$R_{1}$, $R_{n}$-$R_{n+1}$, and $R_{N}$-*D* distances are denoted as $d_{0,1}$, $d_{n,n+1}$, and $d_{N,D}$, respectively. Furthermore, the *S*-*E* and $R_{n}$-*E* distances are computed as $d_{0,E}=\sqrt{d_{E}^{2}+d_{Ex}^{2}}$ and $d_{n,E}=\sqrt{d_{E}^{2}+{\left(d_{0,n}-d_{Ex}\right)}^{2}}$, respectively, where $d_{0,n}=\sum_{m=0}^{n-1}d_{m,m+1}$. It was assumed that channels between any two nodes follow a line-of-sight (LOS) model, where each channel coefficient is evaluated by $d^{-\frac{c}{2}}e^{j\theta }$, where *d* is the distance between the nodes, *θ* is a random phase uniformly distributed within [0,2π), and c=3.5 is the path loss exponent [3,6]. In particular, the residual self-interference channels were also assumed to follow the LOS channel model, and the channel gains for $H_{n,n}$ and $H_{n+1,n}$ were set to be *η* dB smaller than those for $h_{0,1}$ for n=1 and those for $H_{n-1,n}$ for n=2,⋯,N. In the following results, we set $\sigma^{2}=-30$ dBm, N=2, $T_{1}=T_{2}=T$, and $d_{0,1}=d_{1,2}=d_{2,D}=100$ m.

For comparison, we also considered the secrecy rates of the HDRs. In the HDR network, the even-indexed relays were assumed to receive the signal from the previous adjacent nodes in the even time slots and to forward the re-encoded signal to the next adjacent nodes in the odd time slots, whereas the odd-indexed relays performed reception in the odd time slots and transmission in the even time slots. When the beamformers of Equations (6) and (7) were used, it was found that the power allocation problem in the HDR network was the same as that in the FDR network for the PSIC in Equation (16), except that the objective function was multiplied by one half because two time slots are required for the reception and transmission of HDRs. Therefore, the OPA of the HDR was obviously the same as that of the FDR for the PSIC, while the secrecy rate of the HDR with OPA was one half of that of the FDR with OPA for the PSIC. In addition, we also evaluated the secrecy rates for the FDRs with equal power allocation (EPA), where $P_{n}=\frac{P}{N+1}$ with n=0,1,⋯,N.

**Figure 2:**
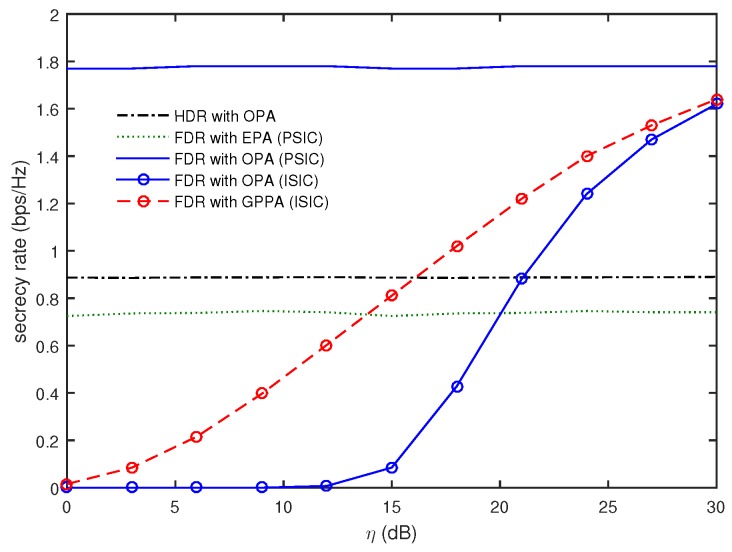
Comparison of secrecy rates as a function of *η* when T=4, $T_{E}=1$, $d_{Ex}=150$ m, $d_{E}=10$ m, and P=60 dBm.

Figure 2 compares the secrecy rates as a function of *η* when T=4, $T_{E}=1$, $d_{Ex}=150$ m, $d_{E}=10$ m, and P=60 dBm. The decrease of *η* implies that the residual self-interference signals became stronger. Note that the FDR for the PSIC and the HDR do not depend on *η*. It was observed that the FDR with OPA for the PSIC provided the best performance. Furthermore, the secrecy rate of the FDR with EPA was worse than that of the HDR with OPA, even though the self-interference signals were perfectly canceled. When we employ OPA for the FDR, even though the residual self-interference exists due to the ISIC, the secrecy rate was found to decrease steeply with the decrease of *η*, and η>20 dB should be guaranteed to provide a better secrecy rate than the HDR with OPA. In this case, the FDR with GPPA outperformed the FDR with OPA and required η>16 dB to provide a better secrecy rate than the HDR with OPA. This confirms that the OPA in Section 3.1 provided the best secrecy rate for the PSIC, while it was not robust for the ISIC. For the ISIC, we have to use the GPPA in Section 3.2 to enhance the secrecy rate.

**Figure 3:**
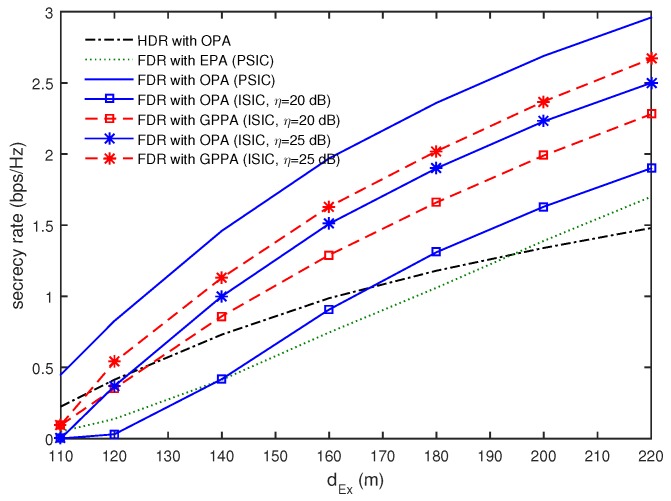
Comparison of secrecy rates as a function of $d_{Ex}$ when T=4, $T_{E}=2$, $d_{E}=10$ m, and P=60 dBm.

Figure 3 shows how the secrecy rates vary with $d_{Ex}$ when T=4, $T_{E}=2$, $d_{E}=10$ m, and P=60 dBm. As expected, the secrecy rate increases as *E* moves away from *S*. It was also confirmed that the FDR with OPA was the best power allocation strategy for the PSIC, while it provided severely degraded performance in all ranges of $d_{Ex}$ for the ISIC. In particular, the FDR with OPA for η=20 dB provided better performance than the HDR with OPA only when $d_{Ex}>170$ m. However, the FDR with GPPA was shown to outperform the HDR with OPA when $d_{Ex}>125$ m. It was noted for the ISIC that the FDR with GPPA was superior to the FDR with OPA in all ranges of $d_{Ex}$.

**Figure 4:**
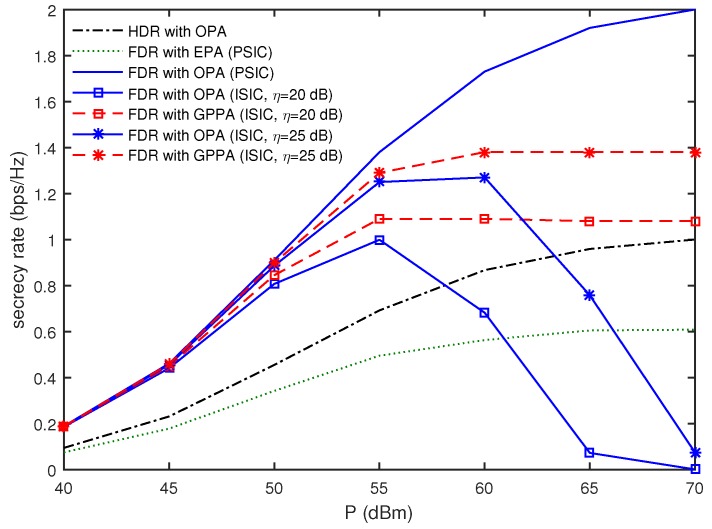
Comparison of secrecy rates as a function of *P* when T=4, $T_{E}=2$, $d_{Ex}=150$ m, and $d_{E}=10$ m.

In Figure 4, the secrecy rates are compared as a function of *P* when T=4, $T_{E}=2$, $d_{Ex}=150$ m, and $d_{E}=10$ m. For the PSIC, the secrecy rate of the FDR with OPA provided the best performance in all ranges of *P* and increased with an increase of *P*, while the FDR with EPA was worse than the HDR with OPA in all ranges of *P*. Next, let us focus on the secrecy rates for the ISIC. As the overall transmit power *P* increases, more transmit power will be assigned to each relay (i.e., $P_{n}$ will increase). When the self-interference channel gain *η* is given, it is evident that the increase of $P_{n}$ results in an increase of the self-interference signal power. For η=20 dB, the secrecy rate of the FDR with OPA was found to increase until P<55 dBm, while it decreased with an increase of *P* and approached zero when P>55 dBm. These observations indicate that the residual self-interference severely affected the secrecy rate performance of the FDR with OPA as the overall transmit power increased. However, it is remarkable that the FDR with GPPA prevented the secrecy rate from decreasing with an increase of *P*, and its secrecy rate performance almost converged when P>55 dBm. For η=25 dB at P>60 dBm, it was observed that the secrecy rate of the FDR with OPA decreased to zero with an increase of *P*, while the FDR with GPPA provided the converged secrecy rate and still outperformed the HDR with OPA.

**Figure 5:**
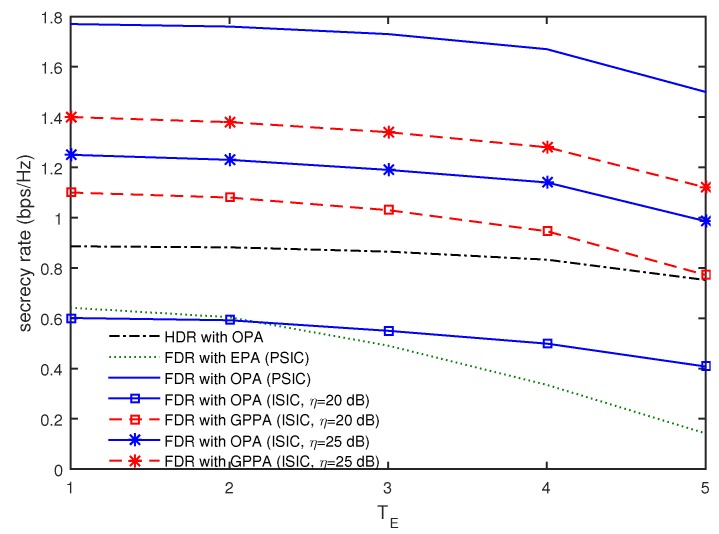
Comparison of secrecy rates as a function of $T_{E}$ when T=6, $d_{Ex}=150$ m, $d_{E}=10$ m, and P=60 dBm.

In Figure 5, we present the secrecy rates according to $T_{E}$ when T=6, $d_{Ex}=150$ m, $d_{E}=10$ m, and P=60 dBm. The secrecy rates are found to decrease with an increase in $T_{E}$. In particular, the FDR with OPA for η=20 dB achieves only 27% to 34% of the secrecy rate of that for the PSIC and is even worse than the HDR with OPA in all ranges of $T_{E}$. Meanwhile, the FDR with GPPA of η=20 dB achieves 51% to 62% of the secrecy rate of the FDR with OPA for the PSIC. For η=25 dB, it is also seen that the FDR with OPA achieves 65% to 71% of the secrecy rate of that for the PSIC, whereas the FDR with GPPA achieves 74% to 79% in all ranges of $T_{E}$. This also confirms that the FDR with OPA is vulnerable to the ISIC and that the GPPA scheme can be utilized to enhance the secrecy rates for the ISIC even in the presence of multiple eavesdroppers.

## 5. Conclusions

In this study, we investigated the secrecy rate of multi-hop DF FDR networks under the overall transmit power constraint when the cooperative beamformer at each individual hop was designed to null out the signals at the eavesdroppers. Using the KKT conditions, we proved that the OPA for PSIC to maximize the achievable secrecy rate was obtained when the overall transmit power constraint and the DF relaying constraints held with equality, which depended on the channel conditions between the source, FDRs, and destination. The channel conditions for the eavesdroppers only influenced whether positive secrecy rates could be achieved. In the case where residual self-interference signals existed owing to the ISIC, we also proposed a suboptimal GPPA scheme to maximize the lower bound of the achievable secrecy rate. From numerical results, we found that the FDR with OPA for PSIC doubled the secrecy rate of the conventional HDR with OPA, while it was vulnerable to the residual self-interference power. For the ISIC, the GPPA scheme was shown to significantly enhance the immunity to the residual self-interference power.

multiple

## Appendix A

In Equation (4), we use the fact that ${\left|h_{n,n+1}^{\left(t\right)}\left(I_{T_{n}}-P_{n,E}\right){\tilde{w}}_{n}\right|}^{2}=\text{tr}\left(H^{\left(t\right)}{\tilde{W}}_{n}\right)$, where tr(.) denotes trace operations, ${\tilde{W}}_{n}={\tilde{w}}_{n}{\tilde{w}}_{n}^{†}$, and

(A1) $$H^{\left(t\right)}={\left(h_{n,n+1}^{\left(t\right)}\left(I_{T_{n}}-P_{n,E}\right)\right)}^{†}h_{n,n+1}^{\left(t\right)}\left(I_{T_{n}}-P_{n,E}\right).$$

Then, the optimization problem in Equation (4) can be rewritten as

(A2) $$\begin{array}{ccc} \max_{{\tilde{W}}_{n}}\min_{t=1,\cdots ,T_{n+1}} & & \text{tr}(H^{\left(t\right)}{\tilde{W}}_{n}),\\ s.t. & & \text{tr}\left({\tilde{W}}_{n}\right)=1,\;{\tilde{W}}_{n}⪰0,\\ & & \text{rank}\;{\tilde{W}}_{n}=1, \end{array}$$

where ${\tilde{W}}_{m}⪰0$ indicates that ${\tilde{W}}_{m}$ must be a symmetric positive semidefinite matrix. Using semidefinite relaxation [34,35], we drop the rank constraint in Equation (A2). Then, we can rewrite Equation (A2) as [22]

(A3) $$\begin{array}{ccc} \max_{{\tilde{W}}_{n},\tau } & & \tau ,\\ s.t. & & \text{tr}\left(H^{\left(t\right)}{\tilde{W}}_{n}\right)\geq \tau ,\;\;t=1,\cdots ,T_{n+1},\\ & & \text{tr}\left({\tilde{W}}_{n}\right)=1,\;\;{\tilde{W}}_{n}⪰0. \end{array}$$

We convert the inequality constraints in Equation (A3) to the equality constraints to obtain

(A4) $$\begin{array}{cc} \min_{{\tilde{W}}_{n},\tau ,s_{t}} & -\tau ,\\ \;s.t. & -\tau -s_{t}+\mathrm{vec}{\left({\tilde{W}}_{n}^{T}\right)}^{T}\mathrm{vec}\left(H^{\left(t\right)}\right)=0,\\ & \tau \geq 0,\;\;s_{t}\geq 0,\;\;t=1,\cdots ,T_{n+1},\\ & \text{tr}\left({\tilde{W}}_{n}\right)=1,\;\;{\tilde{W}}_{n}⪰0, \end{array}$$

which can be solved by SeDuMi [36] and Yalmip [37]. When the solution of Equation (A4), $W_{n}^{★}$, is of rank one, its principal eigenvector can be used as ${\bar{w}}_{n}$. If the rank of $W_{n}^{★}$ is higher than one, we employ a randomization technique [28]. In this study, we eigendecompose $W_{n}^{★}$ as $W_{n}^{★}=U\Lambda U^{†}$ and generate a set of candidate weight vectors, ${\overset{ˇ}{w}}_{k}=U\Lambda^{1/2}v_{k}$, where each entry of $v_{k}$ is $e^{j\theta }$ and *θ* is independently and uniformly distributed on [0,2π). Then, we choose the best result to provide the greatest $\min_{t}{\left|h_{n,n+1}^{\left(t\right)}\left(I_{T_{n}}-P_{n,E}\right){\overset{ˇ}{w}}_{k}\right|}^{2}$.

## Appendix B

The KKT conditions for Equation (16) are given as [22]

(B1) $$\begin{array}{c} \frac{\alpha_{0,E}}{1+\alpha_{0,E}P_{0}}-\alpha_{0,1}\mu_{0}+\mu_{N}=0, \end{array}$$

(B2) $$\begin{array}{c} -\alpha_{n,n+1}\mu_{n}+\mu_{N}=0,\;\;n=1,\cdots ,N-1, \end{array}$$

(B3) $$\begin{array}{c} -\frac{\alpha_{N,D}}{1+\alpha_{N,D}P_{N}}+\alpha_{N,D}\sum_{n=0}^{N-1}\mu_{n}+\mu_{N}=0, \end{array}$$

(B4) $$\begin{array}{c} \alpha_{N,D}P_{N}-\alpha_{n,n+1}P_{n}\leq 0,\;\;n=0,\cdots ,N-1, \end{array}$$

(B5) $$\begin{array}{c} \sum_{n=0}^{N}P_{n}-P\leq 0, \end{array}$$

(B6) $$\begin{array}{c} \mu_{n}\left(\alpha_{N,D}P_{N}-\alpha_{n,n+1}P_{n}\right)=0,\;\;n=0,\cdots ,N-1, \end{array}$$

(B7) $$\begin{array}{c} \mu_{N}\left(\sum_{n=0}^{N}P_{n}-P\right)=0, \end{array}$$

(B8) $$\begin{array}{c} \mu_{n}\geq 0,\;\;n=0,\cdots ,N. \end{array}$$

It is seen that Equation (B2) yields $\mu_{n}=\frac{\mu_{N}}{\alpha_{n,n+1}}$ with n=1,⋯,N−1. Since $\mu_{N}\geq 0$ in Equation (B8), let us consider two cases with $\mu_{N}=0$ and $\mu_{N}>0$.

When $\mu_{N}=0$, we have $\mu_{n}=0$ with n=1,⋯,N−1. In this case, the KKT conditions in Equations (B1)–(B8) are simplified as follows:

(B9) $$\begin{array}{c} \frac{\alpha_{0,E}}{1+\alpha_{0,E}P_{0}}-\alpha_{0,1}\mu_{0}=0, \end{array}$$

(B10) $$\begin{array}{c} -\frac{\alpha_{N,D}}{1+\alpha_{N,D}P_{N}}+\alpha_{N,D}\mu_{0}=0, \end{array}$$

(B11) $$\begin{array}{c} \alpha_{N,D}P_{N}-\alpha_{n,n+1}P_{n}\leq 0,\;\;n=0,\cdots ,N-1, \end{array}$$

(B12) $$\begin{array}{c} \sum_{n=0}^{N}P_{n}-P\leq 0, \end{array}$$

(B13) $$\begin{array}{c} \mu_{0}\left(\alpha_{N,D}P_{N}-\alpha_{0,1}P_{0}\right)=0, \end{array}$$

(B14) $$\begin{array}{c} \mu_{0}\geq 0. \end{array}$$

In Equations (B9) and (B10), it is found that

(B15) $$\mu_{0}=\frac{\alpha_{0,E}/\alpha_{0,1}}{1+\alpha_{0,E}P_{0}}=\frac{1}{1+\alpha_{N,D}P_{N}}>0.$$

Since $\mu_{0}>0$, we have $\alpha_{N,D}P_{N}=\alpha_{0,1}P_{0}$ in Equation (B13). Substituting it into Equation (B15), we found that $\frac{\alpha_{0,E}}{1+\alpha_{0,E}P_{0}}=\frac{\alpha_{0,1}}{1+\alpha_{0,1}P_{0}}$ should be satisfied. This is guaranteed only when $\alpha_{0,E}=\alpha_{0,1}$, which results in $\alpha_{0,E}P_{0}=\alpha_{N,D}P_{N}$. Then, it is obvious that the secrecy rate becomes zero in Equation (16).

Now, we consider the case with $\mu_{N}>0$, which yields $\mu_{n}>0$ with n=1,⋯,N−1. The KKT conditions in Equations (B1)–(B8) are written as

(B16) $$\begin{array}{c} \frac{\alpha_{0,E}}{1+\alpha_{0,E}P_{0}}-\alpha_{0,1}\mu_{0}+\mu_{N}=0, \end{array}$$

(B17) $$\begin{array}{c} -\frac{1}{1+\alpha_{N,D}P_{N}}+\mu_{0}+\left(\sum_{n=1}^{N-1}\frac{1}{\alpha_{n,n+1}}+\frac{1}{\alpha_{N,D}}\right)\mu_{N}=0, \end{array}$$

(B18) $$\begin{array}{c} \alpha_{N,D}P_{N}-\alpha_{0,1}P_{0}\leq 0,\;\;n=0,\cdots ,N-1, \end{array}$$

(B19) $$\begin{array}{c} \alpha_{N,D}P_{N}=\alpha_{n,n+1}P_{n},\;\;n=1,\cdots ,N-1, \end{array}$$

(B20) $$\begin{array}{c} \sum_{n=0}^{N}P_{n}=P, \end{array}$$

(B21) $$\begin{array}{c} \mu_{0}\left(\alpha_{N,D}P_{N}-\alpha_{0,1}P_{0}\right)=0, \end{array}$$

(B22) $$\begin{array}{c} \mu_{0}\geq 0. \end{array}$$

In Equation (B16), one can find that $\mu_{0}>0$ is always true when $\mu_{N}>0$, which yields

(B23) $$\alpha_{N,D}P_{N}=\alpha_{0,1}P_{0}.$$

From Equations (B19), (B20) and (B23), the optimal power allocation is found to satisfy

(B24) $$\begin{array}{c} \alpha_{N,D}P_{N}=\alpha_{n,n+1}P_{n},\;\;n=0,\cdots ,N-1,\;\\ \;\sum_{n=0}^{N}P_{n}=P, \end{array}$$

which implies that the DF relaying constraints and the overall power constraint hold with equality. The solution of Equation (B24) is given in Equation (17). In order to confirm the validity of the solution, we have to check that $\mu_{N}>0$. From Equations (B16) and (B17), we obtain

(B25) $$\mu_{N}=\frac{\gamma (\alpha_{0,1}-\alpha_{0,E})}{\alpha_{0,1}\left(1+\alpha_{0,1}P_{0}\right)\left(1+\alpha_{0,E}P_{0}\right)},$$

where *γ* is defined in Equation (18). It is observed that the condition of $\alpha_{0,1}>\alpha_{0,E}$ guarantees $\mu_{N}>0$.

In the above derivation, we have found that the two cases $\mu_{N}=0$ and $\mu_{N}>0$ correspond to the channel conditions $\alpha_{0,1}=\alpha_{0,E}$ and $\alpha_{0,1}>\alpha_{0,E}$, respectively. When $\alpha_{0,1}<\alpha_{0,E}$, we have the relation of

(B26) $$\alpha_{N,D}P_{N}\leq \alpha_{0,1}P_{0}<\alpha_{0,E}P_{0},$$

owing to the DF relaying constraint. In this case, it is obviously impossible to achieve positive secrecy rates.
